# Beyond Tumors: Gastric Syphilis Emulating a Gastric Neoplasia

**DOI:** 10.3390/medicina60040629

**Published:** 2024-04-13

**Authors:** Claudia Guerrero Muñoz, Andres Castañeda Agredo, Maria Jose Romero Valle, Andrés Mauricio Silva Silva, Marta León del Campo, Claudia Azpirtarte Sánchez, Marta Domínguez Fraga, Manuel Vicente Milán Pilo, Benjamin Arturo Polo Lorduy, Agustina González Guirado, María Jesus Martín Relloso, Paloma Sánchez-Fayos Calabuig, Orencio Bosch Esteva

**Affiliations:** 1 Department of Gastroenterology, Fundación Jiménez Díaz University Hospital, 28040 Madrid, Spain; claudia.guerrero@quironsalud.es (C.G.M.); mjromero@fjd.es (M.J.R.V.); andres.silva@quironsalud.es (A.M.S.S.); mvmilan@fjd.es (M.V.M.P.); bpolo@fjd.es (B.A.P.L.); agguirado@fjd.es (A.G.G.); mjmartin@fjd.es (M.J.M.R.); psanchez@quironsalud.es (P.S.-F.C.); obosch@quironsalud.es (O.B.E.); 2 Department of Pathology, Fundación Jiménez Díaz University Hospital, 28040 Madrid, Spain; 3 Department of Ophthalmology, Fundación Jiménez Díaz University Hospital, 28040 Madrid, Spain; 4 Department of Radiology, Fundación Jiménez Díaz University Hospital, 28040 Madrid, Spain

**Keywords:** endoscopy, gastric cancer, syphilis

## Abstract

We present the case of a 35-year-old male with a first-degree family history of gastric cancer (his father was diagnosed at the age of 45), who was presumed to have gastric cancer himself when evaluating the features of his upper endoscopy performed after hematemesis. Surprisingly, no cancer cells were found in the biopsies. Thanks to a different diagnostic suspicion subsequent to performing a full clinical history, a more favorable diagnosis was reached: gastric syphilis.

## 1. Introduction

Gastric syphilis is a sexually transmitted entity with a rising incidence [1]. According to the World Health Organization (WHO), there is an estimated cumulative incidence of seven million cases annually in adults aged 15–49, with a higher prevalence in men engaged in homosexual relationships. Syphilis remains one of the leading causes of sexually transmitted diseases that can be treated with antibiotics, which are sometimes unavailable, contributing to the ongoing spread of the disease. 

The Syphilis infection is caused by the bacterium Treponema pallidum, manifesting in three distinct stages over time. Initially, it involves the development of an ulcerated lesion in the affected area, followed by systemic involvement, and eventually, a final phase characterized by organic damage, typically occurring many years after the primary infection. Gastric involvement is rare, accounting for only 1% of presentations, predominantly in the secondary stage, and can mimic other diseases, such as gastric cancer, lymphomas, or nodal inflammatory pseudotumors, which are serious diseases requiring early treatment [2,3].

In this article, we explore the case of a patient with gastric syphilis initially thought to be malignant. The objective is to shed light on the diagnostic challenges posed by this rare manifestation, especially when mimicking tumors. The increasing incidence of syphilis and its potential to imitate malignancy highlight the necessity for heightened awareness among clinicians and healthcare providers.

## 2. Case Description

We present the case of a 35-year-old male who was admitted to our hospital’s Emergency Department for evaluation after experiencing two episodes of hematemesis of approximately 200 and 300 mL each within the past 24 h. The patient reported a 3-week history of moderate-to-severe, continuous, and oppressive epigastric pain that worsened towards the end of the day, unresponsive to any conventional pain reliever, omeprazole, and levosulpiride. Additionally, he noted changes in bowel habits, including decreased frequency, bloating, and early satiety after food or water intake, followed by an unintentional weight loss of 12 kg.

He denied changes in dietary habits, former or current use of alcohol, tobacco, nonsteroidal anti-inflammatory drugs, corticosteroids, or herbal supplements. Furthermore, he denied similar previous episodes but had a family history of gastric cancer; his father was diagnosed at the age of 45.

Physical examination was normal with a normal digital rectal exam. Blood tests indicated microcytic hypochromic anemia, with a hemoglobin drop from 14.7 g/dL to 12.7 g/dL in the first 24 h. Other results included a normal BUN-to-creatinine ratio, an elevated INR of 1.88, a Quick index of 43%, and a prolonged prothrombin time of 20.4%.

**Figure 1:**
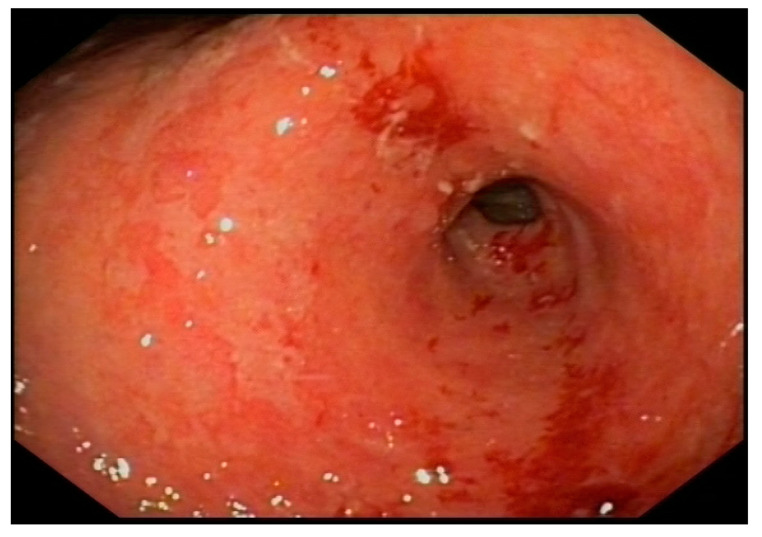
Erythematous and edematous antrum with an ulcerated lesion on the lesser curvature.

With suspected upper gastrointestinal bleeding (Glasgow—Blatchford bleeding score of three), the patient received intravenous Proton Pump Inhibitors (PPIs) and fluid resuscitation. An urgent gastroscopy revealed a thickened, granular, and irregular mucosa on the incisura angularis, with fibrin remnants and a slightly depressed center containing adherent clots (Figure 1). No other ulcers or active lesions were observed. The patient underwent epinephrine (1:10,000) and aethoxysklerol injections, showing no active bleeding. The mucosa of the antrum, extending along the lesser curvature and distal body, presented with edema, erythema, and friability, with a denuded appearance. Only the incisura was biopsied.

**Figure 2:**
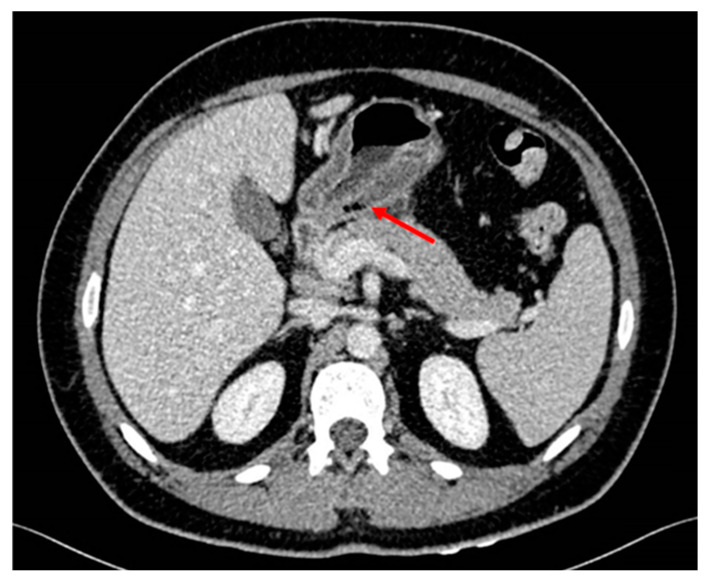
Thickening of the wall of the gastric antrum showing some atypical gas bubbles.

The patient was hospitalized in the Gastroenterology ward with suspicion of malignancy after evaluating endoscopy features. For extension screening, a CT scan was performed, showing wall thickening of the gastric antrum with intramural gas bubbles (Figure 2). Intramural gas could be possibly related to ulceration or to the previous biopsies. There was slight fat stranding as well as some sub-centimeter and non-specific lymph nodes. No signs of distal spreading were seen on the thorax or the rest of the abdomen. Tumor markers were negative.

**Figure 3:**
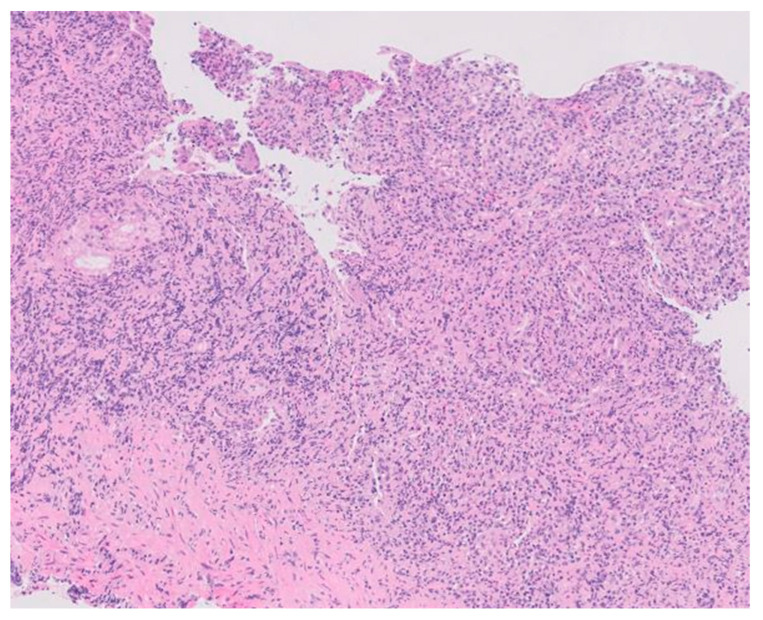
Histopathology of the gastric lesions revealing dense and diffuse lymphoplasmacytic and histiocytic inflammation in the lamina propria with effacement of the normal architecture (hematoxylin-eosin staining, ×100).

A second programmed endoscopy was performed to take more biopsies of the rest of the gastric wall and re-evaluate the mucosa after 48 h of PPI continuous intravenous infusion. The subcardial area, fornix, and the proximal body had no significant abnormalities. The lesser curvature of the distal body, incisura angularis, and antrum showed a thickened erythematous mucosa, presenting ulcerated and friable areas and bleeding remains. Some mucosal areas had a neoplastic appearance. Multiple biopsies were taken with a harder feeling than normal, suggesting acute erosive gastropathy of a probable neoplastic etiology (Figure 3).

A subsequent phone call from the national blood transfusion donation center informed the patient of a positive syphilis result from a donation three weeks earlier. Despite previous negative serologies in April 2014, the patient, in a stable relationship for five years, confirmed previous sexual intercourse using barrier methods.

**Figure 4:**
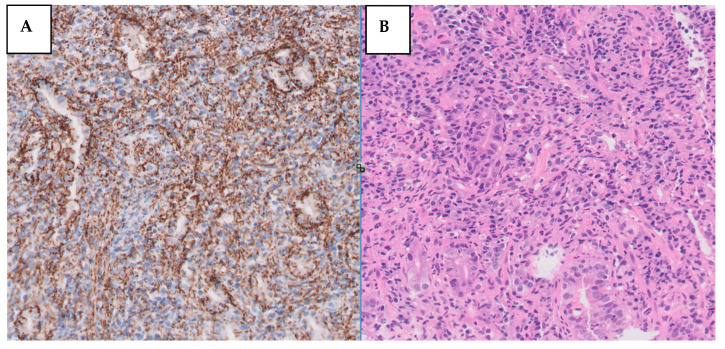
(**A**) Syphilitic involvement of the stomach: dense and mixed mucosal infiltrate. (**B**) Immunohistochemical staining for *T. pallidum* showing numerous spirochetes (×400).

Confirmed syphilis infection prompted treatment with a single dose of 2.4 million units of intramuscular penicillin. Serologies for HIV and hepatitis C were negative, with confirmed protection against HAV and HBV. Gastric tissue biopsy results revealed ulcerated mucosa with a dense inflammatory infiltrate, while immunohistochemistry detected numerous Treponema pallidum spirochetes (Figure 4). Helicobacter pylori bacilli were also identified. The final diagnosis was syphilitic gastritis.

**Figure 5:**
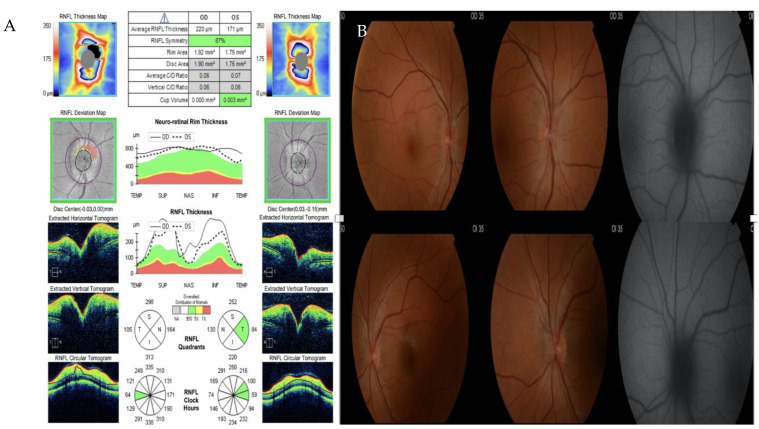
(**A**) Optic coherence tomography showing thickening of all retinal layers. (**B**) Retinography with bilateral papilledema.

During hospitalization, our patient experienced bilateral myodesopsia. An ophthalmological examination found bilateral papilledema without any indications of ocular syphilis (Figure 5). A brain magnetic resonance imaging scan showed no significant intracranial abnormalities and a normal neurological examination was conducted. A lumbar puncture was performed, revealing cerebrospinal fluid (CSF) with elevated total protein levels of 62 mg/dL, glucose at 58 mg/dL, and a cell count of 53 leucocytes/mm^3^ (80% monocytes and 20% polymorphonucleocytes).

Upon ophthalmological reevaluation, signs of anterior uveitis were identified. Early neurosyphilis was suspected, leading to a prescribed treatment of aqueous penicillin G at 3 to 4 million units intravenously every four hours for 14 days.

A transthoracic echocardiogram was conducted, showing normal results. Conventional treatment with omeprazole, amoxicillin, clarithromycin, and metronidazole was administered, successfully eradicating *H. pylori*.

**Figure 6:**
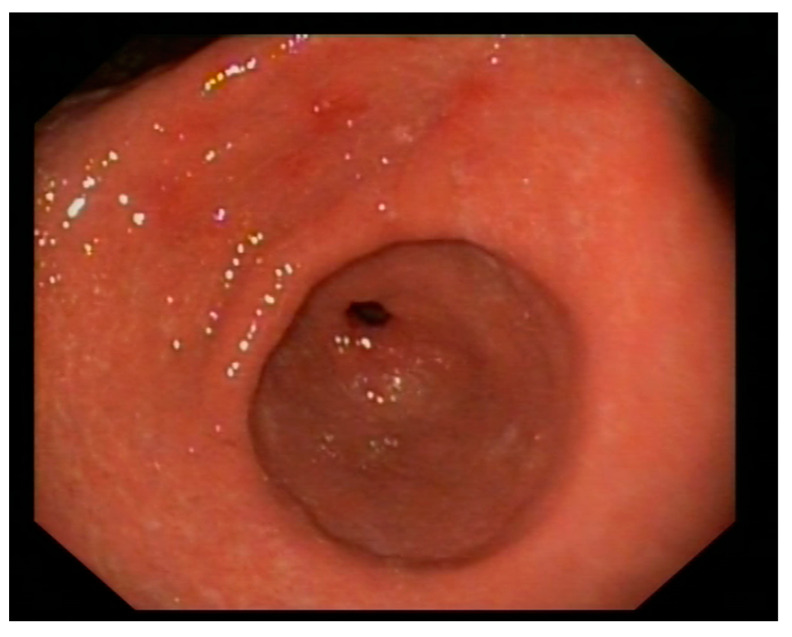
Post-treatment endoscopy showing mucosal healing of the ulcer in the lesser curvature as well as a reduction in the edema with minimal persistence of erythema.

Post-treatment, the patient became asymptomatic and was discharged. Subsequent outpatient clinic follow-up included a control gastroscopy, where a normal mucosa with no signs of ulceration was described, as shown in Figure 6. Multiple biopsies were taken, indicating chronic gastritis without activity and no evidence of metaplasia or dysplasia. The Warthin–Starry stain was negative for *H. pylori*, and immunohistochemistry for Treponema pallidum showed no spirochetes.

## 3. Discussion

Gastric involvement by Treponema pallidum is an uncommon manifestation of this bacteria that can occur in infected patients and has been studied for decades [4,5]. Although syphilis is primarily known to affect the genital organs, it can also affect other systems of the body, including the gastrointestinal system. In this discussion, various aspects related to gastric syphilis are explored.

One aspect to consider is the variety of symptoms that may present in patients with gastric syphilis. The main symptoms include vomiting, epigastric pain, weight loss, early satiety, and anorexia [2,3,4,6,7,8]. These described symptoms are nonspecific, so without clinical suspicion, finding gastric syphilis in an endoscopic procedure is rare and unexpected [4]. Therefore, this can lead to a misdiagnosis or delayed diagnosis.

Gastric involvement in its initial stage usually presents with upper gastrointestinal bleeding secondary to ulceration, followed by a process of obliterative endarteritis characterized by ischemia [7], which can manifest endoscopically as erythema, edema, ulcerations, or nodular mucosa [8,9].

A high index of clinical suspicion is required to consider syphilis as the cause of gastrointestinal symptoms. The initial diagnosis should be made using treponemal and non-treponemal tests, but the final diagnosis is made by taking biopsies demonstrating the presence of Treponema pallidum with immunohistochemical techniques [7,10].

It is important to emphasize that both the symptomatology, imaging tests, and endoscopic findings can mimic other diseases such as tumors, Crohn’s disease, or lymphomas [7,9,11], making their final diagnosis difficult without pathological anatomy tests.

Once diagnosed, treatment is usually favorable with penicillin, leading to the resolution of the lesion and the finding in the blood test in most cases [4,12]. It is crucial to initiate treatment as soon as possible to prevent serious complications, such as gastric perforation or hemorrhage.

The patient did not show any conventional clinical symptoms of syphilis despite an extensive medical history. As we show in the following table, the presence of classic symptoms of primary syphilis was either absent or not noticed by the patients in the past or in the initial evaluation. Considering that gastric syphilis primarily manifests in the secondary stage of the disease, we should look for symptoms of secondary syphilis, which are mainly systemic, such as a generalized rash, which, as observed, was present in some patients from other published clinical cases.

Comparatively, the manifestations for which the patients sought medical attention in the published cases shown in the table are similar (Table 1), with an exact primary moment of infection unknown. The history of gastric neoplasia was only described in our case. Additionally, the predominant site of infection was the gastric antrum, with clinical suspicion of gastric cancer initially, which was ultimately ruled out through pathological anatomy in all cases.

## 4. Conclusions

The diagnostic suspicion of gastric syphilis should be considered in patients with risk factors for sexually transmitted infections who present with nonspecific gastric lesions that may be suspicious for malignancy, especially in young patients with a gastric tumor incidence typically below 10% [13]. This is due to its nonspecific presentation. Our article reveals the need for a thorough study of any gastric lesion to avoid erroneous diagnoses that could lead to inappropriate and possibly exaggerated treatment (for example, surgical resection).

The patient, in our case, presents with a gastric lesion that can easily be confused with a tumor-like growth of malignant etiology. Therefore, it is crucial to conduct a comprehensive evaluation, including specific tests for syphilis, to avoid misdiagnosis and ensure proper patient management.

## Figures and Tables

**Table 1:** Comparison Chart between case reports with similar characteristics.

Comparison Chart between Case Reports						
Case Reports	Time of Infection	Digestive History	Manifestations	Conventional Symptoms	Site of Infection/Suspicion of Gastric Cancer	Final Diagnosis
Guerrero C, Castañeda A et al.	Unknown	Gastric cancer diagnosed in his father at the age of 45	Weight loss, hematemesis, epigastric pain, early satiety	No	Antrum/Yes	Pathological anatomy
Okamoto et al. [5]	Not described	Absent	Epigastric pain, nausea, emesis, weight loss	Yes, Skin Rash	Greater curvature/No	Pathological anatomy
Lan YM et al. [2]	Same sexual partner for 1 year but his partner was promiscuous	Absent	Epigastric and mesogastric pain, nausea, weight loss	No	Antrum-pylorus/Yes	Pathological anatomy
Sinagra E et al. [3]	Not described	Absent	Epigastric pain, nausea, anorexia, vomiting, weight loss	Yes, skin rash and lymphadenophaty	Antrum/Yes	Pathological anatomy
Verma R et al. [7]	Not described	Absent	Hematemesis, epigastric pain	No	Antrum/Yes	Pathological anatomy

## Data Availability

No new data were created.
